# Performance of health workers on neonatal resuscitation care following scaled-up quality improvement interventions in public hospitals of Nepal - a prospective observational study

**DOI:** 10.1186/s12913-021-06366-8

**Published:** 2021-04-19

**Authors:** Dipak Raj Chaulagain, Mats Malqvist, Olivia Brunell, Johan Wrammert, Omkar Basnet, Ashish KC

**Affiliations:** 1 Department of Women’s and Children’s Health, Uppsala University, Uppsala Global Health Research on Implementation and Sustainability (UGHRIS), Dag Hammarskjölds väg 14B, 75185 Uppsala, Sweden; 2 Golden Community, Lalitpur, Nepal; 3 Society of Public Health Physicians Nepal (SOPHPHYN), Kathmandu, Nepal

**Keywords:** Quality improvement, Intrapartum hypoxic events, Neonatal resuscitation, Scale-up, Bag and mask ventilation, Additional stimulation, Clearing of airways, Nepal

## Abstract

**Background:**

High-quality resuscitation among non-crying babies immediately after birth can reduce intrapartum-related deaths and morbidity. Helping Babies Breathe program aims to improve performance on neonatal resuscitation care in resource-limited settings. Quality improvement (QI) interventions can sustain simulated neonatal resuscitation knowledge and skills and clinical performance. This study aimed to evaluate the effect of a scaled-up QI intervention package on the performance of health workers on basic neonatal resuscitation care among non-crying infants in public hospitals in Nepal.

**Methods:**

A prospective observational cohort design was applied in four public hospitals of Nepal. Performances of health workers on basic neonatal care were analysed before and after the introduction of the QI interventions.

**Results:**

Out of the total 32,524 births observed during the study period, 3031 newborn infants were not crying at birth. A lower proportion of non-crying infants were given additional stimulation during the intervention compared to control (aOR 0.18; 95% CI 0.13–0.26). The proportion of clearing the airway increased among non-crying infants after the introduction of QI interventions (aOR 1.23; 95% CI 1.03–1.46). The proportion of non-crying infants who were initiated on BMV was higher during the intervention period (aOR 1.28, 95% CI 1.04–1.57) compared to control. The cumulative median time to initiate ventilation during the intervention was 39.46 s less compared to the baseline.

**Conclusion:**

QI intervention package improved health workers’ performance on the initiation of BMV, and clearing the airway. The average time to first ventilation decreased after the implementation of the package. The QI package can be scaled-up in other public hospitals in Nepal and other similar settings.

## Background

Intrapartum hypoxic events annually account for one-quarter of 2.5 million neonatal deaths worldwide [1, 2]. The appropriate resuscitation action by health workers within one minute after birth (golden minute) can prevent these deaths [1, 3, 4]. Also, immediate and proper resuscitation care can reduce associated long-term complications like cerebral palsy, epilepsy, and learning difficulties in surviving infants [5]. The Helping Babies Breathe (HBB) program was initiated to improve health workers’ performance on basic resuscitation care in resource-limited settings [6]. Studies show that the HBB program improves the timely initiation of bag and mask ventilation (BMV) and reduce early neonatal mortality and stillbirths [7–9]. Despite these efforts, low-and-middle income countries are still struggling to improve the performance of health workers and to reduce intrapartum-related deaths at scale [10–12]. When HBB training is implemented without additional implementation strategies, the neonatal resuscitation knowledge and skills do not last long and do not translate into clinical performance [13–15]. Quality improvement (QI) interventions including frequent skill drills, continuous mentoring, and frequent training sessions have the potential to sustain and transfer simulated neonatal resuscitation knowledge and skills into clinical performance [12, 14, 16, 17].

Health workers’ anxiety and fear, difficulties assessing the infant’s condition, and providing appropriate clinical response at the time of delivery often delay the initiation of BMV [18]. A study in Tanzania reported better neonatal resuscitation practice in simulated settings 7 months after one-day HBB training, but this improvement did not translate into clinical performance [13]. However, a systematic review and meta-analysis reported an improved initiation of BMV within 1 min of birth after the implementation of the HBB program [7]. Furthermore, the resuscitation performances are improved with health workers’ ability to monitor labour adequately, preparing resuscitation equipment before delivery, teamwork, and frequent resuscitation training [18].

Nepal is committed to improve the quality of neonatal care to reduce the current neonatal mortality rate (NMR) of 21 per 1000 live births to 11 per 1000 live births by 2035 [19, 20]. In between 2001 and 2016, Nepal observed an annual rate of reduction (ARR) of 4.0% for neonatal mortality with a wide disparity among the socioeconomic groups; ARR of 6.3% among the wealthiest and 2.0% among the poorest quintile [21]. To achieve the Sustainable Development Goal (SDG) of reducing NMR proportionately among all socio-economic groups, the inequality in NMR reduction should be addressed through a multi-sectoral approach focusing on gaps in quality of care [19, 21]. The gaps in neonatal resuscitation are mainly associated with health system readiness on health workforce, essential commodities, and service delivery [22, 23]. Persisting gaps in neonatal resuscitation performance warrant the continuous search for sustainable strategies and approaches to improve gains in neonatal care outcomes [24, 25].

A prospective observational study in a tertiary maternity hospital in Nepal demonstrated that HBB with QI package can reduce intrapartum stillbirth and first-day neonatal mortality, and can improve BMV within 1 min of birth [16]. Based on this study and recent evidence on QI interventions in other settings, the Ministry of Health and Population together with the study team developed a QI intervention package [26]. The QI package aimed to improve meso (hospital management) and micro (health worker) level performances [27]. The package was based on a plan-do-study-act (PDSA) approach [28] with three major strategies; i) facilitation, ii) training and iii) audit and feedback [29]. We scaled up this QI package in 12 public hospitals in Nepal. Out of those 12 hospitals, the performance of health workers on neonatal resuscitation was independently observed and recorded in four high-volume hospitals. This study aimed to evaluate the effect of this scaled-up QI package on the performance of health workers on basic neonatal resuscitation care in those hospitals.

## Methods

We report this study using the Strengthening the Reporting of Observational Studies in Epidemiology (STROBE) checklist (S1 STROBE checklist).

### Study design

This was a prospective observational cohort study nested within the stepped wedge cluster randomized controlled trial for Nepal Perinatal Quality Improvement Project (NePeriQIP) [26]. Performances of health workers on basic neonatal care; additional stimulation, clearing the airway, and bag and mask ventilation (BMV) were analysed before and after the introduction of the QI interventions.

### Study settings

Out of 12 hospitals under NePeriQIP, we conducted this study in four high-volume hospitals with > 8000 deliveries per year at the time of enrolment in the study. All of the participating hospitals were secondary referral hospitals situated along the flatlands and had specialized sick newborn care services. The hospitals differed in service coverage and population they serve in terms of language, ethnicity, and religion.

### Quality improvement intervention package

We introduced the QI package in all participating hospitals in a similar fashion. At the start, the hospital management team was oriented on the QI package. The hospital management then selected four in-hospital QI facilitators from among the paediatricians, medical officers, and nurses. External mentors were selected and recruited by the study team to support in-hospital QI facilitators in implementing the QI package in the hospital. In-hospital QI facilitators along with the external mentors received a seven-day master training of trainers (MTOT) on the QI package. Following the MTOT, QI facilitators assessed the readiness and availability of perinatal care services using a checklist in their respective hospitals. A two-day bottleneck analysis workshop was organized at each hospital to analyse the findings of service readiness and availability assessment. After this, the health workers involved in perinatal care in each hospital received a three-day basic training on the QI package. The training contents included HBB package together with QI interventions. Each hospital was provided with the HBB job aid, self-assessment checklists, HBB mannequin set for the skill check, scoreboards, and weekly PDSA review meeting notes. After the basic training, the QI facilitators initiated weekly PDSA meetings involving health workers providing perinatal care. The health workers started daily skill-check on BMV using the mannequin, self-assessment of preparation for resuscitation before every delivery, and for those requiring BMV. Besides, the QI facilitators started updating the scoreboard installed in the delivery room. Six months after the basic training, health workers received a one-day refresher course on QI interventions with a focus on HBB.

### Participants

The women in labour at ≥22 weeks’ gestation who gave consent were eligible for the study. We excluded women whose foetus had no fetal heart sound before admission to labour, those referred for caesarean delivery, and those who were referred to other facilities. We enrolled all eligible women who gave birth to a non-crying infant in this study.

### Sample size

There was no a priori estimation of sample size and we used the larger study sample calculation. All of the eligible women who provided consent and gave birth to a non-crying infant were included in the study.

### Variables

Following were the main outcome variables in the study;

- Additional stimulation* - Additional stimulation is defined as rubbing the back of the infants who do not cry spontaneously even after thorough drying. The back of the infants is rubbed gently two to three times to establish spontaneous breathing.
- Clearing the airway* - Clearing the airway is defined as removing the secretions from the airway if the infant is not breathing and secretions are seen on the airway. The secretions are removed by wiping the mouth and nose with a cloth or gently suctioning the mouth and nose with bulb suction or suction tube.
- **Initiation of BMV** - Starting ventilation with bag and mask for those babies who do not establish spontaneous breathing even after additional stimulation and clearing of airways.
- **Initiation of BMV within 1 min** - Starting ventilation to non-crying infants before 60 s after birth.
- **Time taken to initiate first ventilation** - The time taken after birth to start ventilation using bag and mask.
- **Appropriate Ventilation rate** - Ventilation at the rate of 30 to 50 breaths per minute.

Other variables included; rising of the chest after each ventilation, repositioning of the head if no rise in the chest was observed, and assessment of heart rate after 1 min of ventilation.

* For comparison of the first two outcome variables (additional stimulation and clearing the airway) between control and intervention period, the non-crying infants were further categorized into two sub-groups; a) non-crying and non-breathing (NCNB), and b) non-crying but breathing (NCB). Further categorization was done because non-crying at birth does not necessarily mean non-breathing. However, non-crying but breathing infants also require additional care because breathing may deteriorate sometimes after birth [30].

### Data collection

The data collection team comprised of nurses with experience in nursing management and research. In each hospital, the team consisted of eight data collectors who worked on a rotation basis to ensure observation of all eligible participants. A seven-day training was organized for data collectors to simulate using data collection forms. The data collection team was managed by a coordinator to ensure an effective data collection process. A standard operating procedure (SOP) was developed to guide data collectors to ensure completeness, consistency, and accuracy of data. The study team members from Kathmandu visited the participating hospitals frequently to ensure a smooth collection of data. The data collection coordinator met with the study team every week online to discuss the progress in data collection. Any discrepancy on clinical observation recorded in the form was identified and corrected timely. We collected data in paper formats from all hospitals through direct observation of clinical performance. The pre-testing of the form was done in a tertiary hospital in Kathmandu before organizing training of data collectors.

### Data management

The data collection coordinator sent completed forms weekly to the central research office in Kathmandu. After rechecking for completeness and consistency, the data were transferred into an electronic database, Census and Survey Processing System (CSPro) by a team of independent data entry officers. The forms were indexed for respective hospitals before being entered into the database. An independent investigation team performed data quality assessment using pre-developed SOP quarterly and provided feedback to data collection teams accordingly.

### Statistical analyses

Descriptive statistics were calculated for background characteristics of the non-crying infants for the control and intervention period. The proportion with 95% confidence intervals and *p*-values were calculated for each outcome variable. Chi-square test was performed for comparison of outcome variables between the control and intervention period. The odds ratio with 95% confidence interval and *p*-values were calculated for each outcome variable. Logistic regression analysis was performed to adjust three possible confounding background variables (preterm birth, assisted breech delivery, and meconium-stained amniotic fluid at birth*)* that were different between the control and intervention period. A time plot was created to show the cumulative monthly average time to first ventilation with bag and mask. A *p*-value of less than 0.05 was considered statistically significant. Missing data were excluded from the analysis for additional stimulation, clearing the airway, Initiation of BMV, Initiation of BMV within 1 min, appropriate ventilation rate, selection of correct mask for ventilation, rising of the chest after each ventilation, repositioning of the head, and assessment of heart rate. The Statistical Package for the Social Sciences (SPSS) version 25.0 was used for all analyses.

### Trial registration

This study was a part of the larger Nepal Perinatal Quality Improvement Project (NePeriQIP) with International Standard Randomised Controlled Trial Number, ISRCTN30829654, registered 17th May 2017.

## Results

**Fig. 1:**
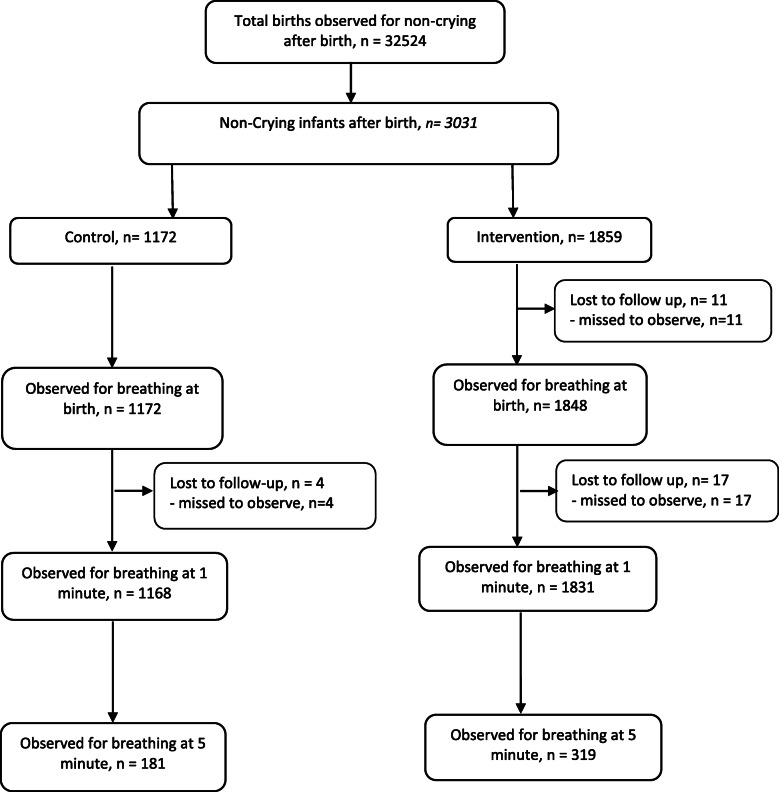
Strengthening the Reporting of Observational Studies in Epidemiology flow diagram for inclusion in the analysis

Out of the total 51,818 deliveries, we observed 32,524 during the period of July 2017 to October 2018. Altogether 3031 infants who were not crying at birth were included in the analysis; 1172 in control and 1859 in the intervention period (Fig. 1). Among those women who delivered non-crying infants, the proportion of assisted breech delivery was found higher during the intervention period (*p* < 0.001), as were the proportion of augmentation of labour (*p* < 0.001), episiotomy performed (*p* = 0.045), infants born preterm (*p* < 0.001) and infants born with meconium-stained amniotic fluid (*p* < 0.001) (Table 1).

**Table 1:** Background characteristics of non-crying infants at birth, *N* = 3031

Characteristics	Control (***N*** = 1172, % with 95% CI)	Intervention (***N*** = 1859, % with 95% CI)	Total (N = 3031, % with 95% CI)	***P*** value
1. Maternal age (Mean ± SD)	23.48 ± 4.11	23.68 ± 4.17	23.60 ± 4.15	0.198
2. Disadvantaged Ethnic group	70.2% (67.5–72.8)	72.0% (69.9–74.1)	71.3% (69.7–72.9)	0.284
3. Illiterate mothers	7.1% (5.5–9.0)	6.4% (5.2–7.8)	6.7% (5.7–7.7)	0.485
4. Sex of the baby				
*Male*	62.0% (59.2–64.8)	64.9% (62.7–67.0)	63.8% (62.0–65.5)	0.113
*Female*	38.0% (35.2–40.8)	35.1% (33.0–37.3)	36.2% (34.5–38.0)	
5. Maternal complication during delivery	1.6% (0.9–2.6)	2.7% (1.9–3.7)	2.3% (1.7–3.0)	0.063
6. Augmentation of labour	49.0% (46.0–51.9)	55.8% (53.4–58.2)	53.1% (51.3–55.0)	***0.000***
7. Abnormal Fetal heart rate during delivery	3% (2.0–4.2)	2.5% (1.7–3.5)	2.7% (2.1–3.5)	0.452
8. Episiotomy performed	48.2% (45.3–51.2)	52.2% (49.6–54.9)	50.5% (48.5–52.4)	***0.045***
9. Mode of delivery				
*Spontaneous vaginal*	89.5% (87.6–91.2)	88.4% (86.9–89.9)	88.8% (87.7–89.9)	0.362
*Instrumental*	9.6% (7.9–11.4)	8.7% (7.4–10.0)	9.0% (8.0–10.1)	0.402
*Assisted breech*	0.9% (0.5–1.7)	2.9% (2.2–3.8)	2.1% (1.7–2.7)	***0.000***
10. Parity				
*Nullipara*	51.7% (48.5–54.9)	50.6% (48.2–53.1)	51.0% (49.1–53.0)	0.603
*Primipara*	30.6% (27.7–33.6)	31.0% (28.7–33.3)	30.8% (29.0–32.6)	0.845
*Multipara*	17.7% (15.4–20.3)	18.4% (16.5–20.4)	18.1% (16.7–19.7)	0.661
11. Low birth weight	22.7% (20.3–25.2)	22.6% (20.7–24.6)	22.6% (21.2–24.2)	0.947
12. Preterm birth	9.6% (8.0–11.5)	16.4% (14.7–18.1)	13.8% (12.6–15.0)	**0.000**
13. Multiple birth	2.4% (1.6–3.4)	3.1% (2.4–4.0)	2.8% (2.3–3.5)	0.233
14. Malformation of newborn infants	1.4% (0.8–2.3)	2.3% (1.5–3.3)	1.9% (1.4–2.5)	0.121
15. Infants with meconium stained amniotic fluid at birth	29.8% (27.2–32.5)	23.7% (21.7–25.7)	26.1% (24.5–27.7)	**0.000**

A lower proportion of non-crying infants were stimulated during the intervention (83.8%) than that in control period (96.7%) (aOR 0.18; 95% CI, 0.13–0.26). Additional stimulation to non-crying and non- breathing infants was lower during the intervention period (aOR 0.28; 95% CI, 0.17–0.43). Additional stimulation to non-crying but breathing infants also decreased during the intervention period (aOR 0.22; 95% CI, 0.12–0.41) (Table 2).

**Table 2:** Performance of health workers on newborn infants requiring basic resuscitation care

	Control, (*N* = 1172, % with 95% CI)	Intervention, (*N* = 1859, % with 95% CI)	OR (95% CI), ***p value***	aOR^**b**^ (95% CI), ***p*** value
1. Additional stimulation to non-crying infants, *(*^a^*n*^*c*^ *= 1169, n*^*i*^ *= 1724)*	96.7% (95.5–97.6)	83.8% (81.9–85.5)	***0.86 (0.84–0.88), p = 0.000***	***0.18 (0.13–0.26), p = 0.000***
2. Additional stimulation to non-crying and non-breathing (NCNB) infants, *(n*^*c*^ *= 297, n*^*i*^ *= 715)*	91.2% (87.4–94.2)	70.1% (66.6–73.4)	***0.76 (0.72–0.81), p = 0.000***	***0.28 (0.17–0.43), p = 0.000***
3. Additional stimulation to non-crying but breathing (NCB) infants, *(n*^*c*^ *= 872, n*^*i*^ *= 1001)*	98.5% (97.5–99.2)	93.6% (91.9–95.0)	***0.95 (0.93–0.96), p = 0.000***	***0.22 (0.12–0.41), p = 0.000***
4. Clearing of airway to non-crying infants, *(n*^*c*^ *= 1166, n*^*i*^ *= 1702)*	68.3% (65.5–70.9)	70.9% (68.7–73.1)	1.03 (0.98–1.09), *p* = 0.129	**1.23 (1.03–1.46),** ***p*** **= 0.017**
5. Clearing of airways to non-crying and non-breathing (NCNB) infants, *(n*^*c*^ *= 297, n*^*i*^ *= 712)*	80.8% (75.9–85.1)	67.3% (63.7–70.7)	***0.83 (0.77–0.89), p = 0.000***	***0.65 (0.45–0.93), p = 0.018***
6. Clearing of airways to non-crying but breathing (NCB) infants *(n*^*c*^ *= 869, n*^*i*^ *= 980)*	64% (60.7–67.2)	73.5% (70.6–76.2)	***1.14 (1.07–1.22), p = 0.000***	***1.59 (1.29–1.96), p = 0.000***
7. Initiated bag and mask ventilation (BMV) to non-crying infants, *(n*^*c*^ *= 1167, n*^*i*^ *= 1732)*	16.0% (14.0–18.3)	19.6% (17.8–21.6)	***1.22 (1.04–1.44), p = 0.013***	***1.28 (1.04–1.57), p = 0.016***

^a^n^c^ = number of cases in the control group, n^i^ = number of cases in the intervention

^b^adjusted for preterm birth, assisted breech delivery, and meconium-stained amniotic fluid at birth

We found an improvement in the performance of health workers on clearing the airway (wiping or suctioning of the mouth and nose) among non-crying infants after implementing the QI package (Table 2). Overall, clearing of the airways of non-crying infants increased during the intervention period compared to control (aOR 1.23; 95% CI, 1.03–1.46). A lower proportion of clearing the airway was observed among non-crying and non-breathing infants during the intervention period (aOR, 0.65; 95% CI, 0.45–0.93). However, clearing the airway of non-crying but breathing infants was higher during the intervention period (aOR 1.59; 95% CI, 1.29–1.96) (Table 2).

We observed improved performance of health workers on a key resuscitation action; initiation of BMV to non-crying infants after QI interventions. The proportion of non-crying infants who were initiated on BMV was higher during the intervention period (aOR 1.28; 95% CI, 1.04–1.57) compared to control (Table 2).

**Fig. 2:**
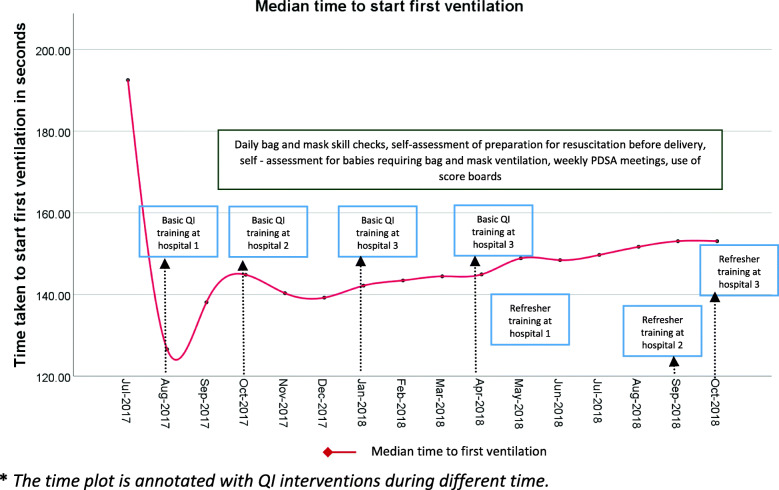
The time plot. The monthly cumulative median time to first ventilation among those infants who were initiated on BMV*

The ventilation-time plot showed a sharp decline in cumulative median time to first ventilation by the end of the second month of the study when QI interventions were started in the first hospital (Fig. 2). The median time increased slightly during the third and fourth months of data collection and again dropped down slightly during November and December 2017. From January 2018 onwards, the median time for first ventilation began to rise slowly and by the end of October 2018, the cumulative median time was found to be 153.07 s. The cumulative median time to initiate ventilation as of the last month of data collection was 39.46 s less compared to the baseline period (Fig. 2).

The proportion of infants who were initiated on BMV within 1 minute did not change before and after the implementation of the QI package (OR 0.83; 95% CI, 0.51–1.33) *(*Table 3*)*. Selection of correct mask for ventilation (OR 0.99; 95% CI, 0.96–1.03) and ventilation at the rate of 30–50 breaths per minute (OR 0.92; 95% CI, 0.80–1.06) remained unchanged before and after the initiation of the intervention. Likewise, no change was observed in rising of the chest after each ventilation attempt (OR 1.03; 95% CI, 0.95–1.13) and repositioning of the head if no rise in the chest was observed (OR 0.98; 95% CI, 0.85–1.13) during the intervention period. Assessment for heart rate after 1 min of initiating BMV also remained the same during control and intervention (OR, 0.91; 95% CI, 0.80–1.04) (Table 3).

**Table 3:** Performance of health workers on ventilation among those infants who were initiated on BMV

	Control, (***N*** = 187, % with 95% CI)	Intervention, (***N*** = 342, % with 95% CI)	Total, (***N*** = 529, % with 95% CI)	OR (95% CI)	***P*** value
1. Initiation of BMV within 1 min	13.4% (8.8–19.1)	11.1% (8.0–14.9)	11.9% (9.3–15.0)	0.83 (0.51–1.33)	0.433
2. Selected correct mask for ventilation, *(n*^*c*^ *= 186, n*^*i*^ *= 340)*	95.7% (91.7–98.1)	95.6% (92.8–97.5)	95.% (93.5–97.2)	0.999 (0.96–1.03)	0.953
3. Ventilated at the rate of 30–50 breaths per minute BMV, *(n*^*c*^ *= 177, n*^*i*^ *= 249)*	67.2% (59.8–74.1)	62.2% (55.9–68.3)	64.3% (59.6–68.9)	0.92 (0.80–1.06)	0.290
4. Chest risen after each ventilation, *(n*^*c*^ *= 185, n*^*i*^ *= 324)*	80% (73.5–85.5)	83% (78.5–86.9)	81.9% (78.3–85.2)	1.03 (0.95–1.13)	0.394
5. Repositioned the head if no rise in chest was observed, *(n*^*c*^ *= 44, n*^*i*^ *= 127)*	86.4% (72.6–94.8)	85% (77.6–90.7)	85.4% (79.2–90.3)	0.98 (0.85–1.13)	0.830
6. Assessed for heart rate after 1 min of ventilation, *(n*^*c*^ *= 183, n*^*i*^ *= 319)*	69.4% (62.2–76.0)	63.6% (58.1–68.9)	65.7% (61.4–69.9)	0.91 (0.80–1.04)	0.190

## Discussion

This is one of the well-evaluated scaled-up studies to assess the performance of health workers on basic neonatal resuscitation in Nepal. The QI package introduced in public hospitals resulted in an increased proportion of initiation of BMV. Additional stimulation to non-crying infants was found to be performed less during the intervention period compared to control. But, we observed a higher proportion of clearing the airway among non-crying infants after initiating QI interventions. The cumulative average time to initiate ventilation decreased slightly during the intervention period.

The HBB focuses on thorough drying, additional stimulation, clearing the airway if required followed by BMV (when required) within 1 min of birth to support non-crying infants to establish spontaneous breathing [6]. The HBB protocol recommends that all non-crying infants should be given additional stimulation that will reduce the number of infants requiring BMV [31]. However, the use of additional stimulation should not result in the delayed initiation of BMV [16]. Less additional stimulation practice after QI interventions in our study differs from the finding of a study conducted in Tanzania that reported an increase in the use of stimulation after HBB training [8]. Similarly, our finding is different from the finding of a recent systematic review and meta-analysis which reported no changes in the use of stimulation after the introduction of HBB [7]. However, the proportion of stimulation in our study during the intervention period is almost similar to the proportion of stimulation after HBB training in Tanzania [8]. One explanation for the decreased rate of stimulation in our study could be that the data collectors might have failed to observe and record the quick rubbing of the back of infants by health workers. As part of the QI intervention, the training sessions instructed health workers on giving quick additional stimulation. Before the QI intervention, the health workers were spending more time on stimulation and suctioning delaying the time to initiate BMV.

Informed by the International Liaison Committee on Resuscitation Consensus on Science and Treatment Recommendations (ILCOR CoSTR 2015), the revised HBB protocol states that suctioning is needed for infants only when they do not start breathing even after drying and secretions are seen in the airway [31]. World Health Organization’s newborn resuscitation guidelines recommend that suctioning is required for those infants who are not breathing on their own and the mouth or nose is full of secretions or infants are born with meconium-stained amniotic fluid [32]. The proportion of clearing the airway among non-crying newborn infants was higher during the intervention period compared to control in our study. A similar study in Tanzania reported the proportion of suctioning increased from 15 to 22% after HBB training [8]. But, The proportion of suctioning decreased from 26.7 to 10% after HBB training in India [33]. Budhathoki S et al. reported no changes in suctioning before and after the introduction of HBB based on the systematic review and meta-analysis of four studies [7]. The proportion of clearing the airway among non-crying infants in our study during the intervention period is much higher than that reported by Goudar et al., Mseomo et al., and KC et al. [8, 16, 33]. However, there is a lack of standard on the desired proportion of non- crying newborn infants to be suctioned. Clearing the airway included wiping of the secretions with a cloth or suctioning of mouth and nose with a bulb or tube in our study, and analyses were performed only for those babies who were not crying at birth. This is the reason why the proportion of “clearing the airway” seemed higher than that reported by other studies. The increase in clearing of airways among non-crying infants can be attributed to different QI interventions introduced in the hospital.

Initiation of BMV is crucial in saving non-crying newborn infants’ life [31]. Our study reveals the QI interventions improve the initiation of BMV to non-crying infants. Our finding differs from the systematic review and meta-analysis which reported no changes in the proportion of initiation of BMV before and after HBB [7]. Our finding also differs from the study in Tanzania which reported the decreased proportion of BMV after HBB training [8]. Similar to our finding, an increased proportion of BMV after HBB training was reported in Sudan [34]. Among the non-crying infants, bag and mask ventilation should ideally be started within 1 min after birth [31]. We did not observe improvement in the proportion of initiation of BMV within 1 min of birth. The inadequate number of health workers available in the delivery room might have affected this performance. Our finding differs from the systematic review and meta-analysis based on the studies in India and Nepal which concluded an increased proportion of BMV initiation within 1 min of birth after HBB training [7, 16, 33]. However, the median time to initiate ventilation after birth in our study dropped by 39.46 s by the last month of data collection. The cumulative median time by the last month of data collection (153.07 s) is however far from the ideal time (golden minute). Further studies are required on how to improve BMV within the golden minute of birth considering the existing contextual factors (number of human resources available, work culture, motivation etc.).

Our findings show QI interventions can result in improved performance of health workers (micro-level) on neonatal resuscitation. The important aspects of improving neonatal resuscitation performance were leadership commitment, improving hospital context, supply of essential equipment, capacity building of health workers, regular technical mentoring, daily bag-and-mask skill checks, preparation for resuscitation before every delivery, and self-assessment of the bag and mask ventilation. Addressing the gaps in the hospital context; number of health workers available, work culture, motivation etc. may result in better performance and outcome of neonatal care in the future.

Our study has two major strengths. First, this is the first multi-center large-scale study to assess the effectiveness of scaled-up QI interventions in improving health workers’ performance on neonatal resuscitation. Second, the QI intervention was initiated in the existing set-up-of public hospitals, without any modifications in structure and management. The participating hospitals represent the existing hospital levels in Nepal, allowing our findings to be fairly generalized to other similar hospital settings.

Our study has some limitations. First, we did not record the outcome of additional stimulation and clearing the airway in terms of spontaneous breathing. The HBB protocol states that most of the non-crying infants initiate spontaneous breathing with these initial steps. Second, it was difficult for data collectors to observe and fill the form on clinical activities of health workers within 1 min. Therefore, the data collectors might have failed to observe and record some of the action steps of health workers especially when there were multiple deliveries at the same time. Third, we did not relate the specific QI tools with the resuscitation performance of health workers. This could have generated deeper insight into the specific QI intervention bundled in the package. Fourth, we did not analyse the outcome of BMV among non-crying infants in terms of spontaneous breathing. Fifth, we did not analyse existing contextual factors of hospitals against the performance of health workers on neonatal resuscitation. Lastly, we could observe only 62% of the total deliveries during the study period that might have some effect of selection bias in our results. We could not observe the remaining deliveries mainly due to the lack of mothers’ consent and due to referral for caesarean delivery. Also, the data collectors failed to observe some of the deliveries because of time constraints.

The input for QI intervention in our study seemed to be resourceful in terms of finance, personnel, and the logistics involved. However, the results indicate that the QI intervention package can be considered effective and feasible for scale-up in other hospitals. The anecdotal feedback from hospital management and health workers, and our observations, suggest that the amount of input provided for the QI intervention was the needed investment for better outcomes in neonatal care.

## Conclusion

We conclude that the QI intervention package is effective to improve the initiation bag and mask ventilation. Clearing the airway among non-crying infants also increased after implementing QI interventions. The average time to start the ventilation decreased after the implementation of the QI package. The QI package can be scaled-up in other public hospitals in Nepal and other similar settings. Further studies are indicated to search for ways for further improving BMV within the golden minute of birth and sustaining the gains of QI interventions.

## Data Availability

The datasets used and/or analysed during the current study are available from the corresponding author on reasonable request.

## Abbreviations

ARR: Annual rate of reduction
BMV: Bag and mask ventilation
CI: Confidence interval
CSPro: Census and Survey Processing System (CSPRO).
HBB: Helping Babies Breathe
ILCOR CoSTR: International Liaison Committee on Resuscitation Consensus on Science and Treatment Recommendations
ISRCTN: International Standard Randomised Controlled Trial Number
MToT: Master training of trainers
NCB: Non-crying but breathing
NCNB: Non-crying and non-breathing
NePeriQIP: Nepal Perinatal Quality Improvement Project
NMR: Neonatal mortality rate
OSCE: Objective structured clinical examination
PDSA: Plan-do-study-act
QI: Quality improvement
SDG: Sustainable Development Goals
SOP: Standard operating procedure
SPSS: Statistical Package for the Social Sciences (SPSS)
